# Abdominoscrotal Hydrocele with Intestinal Malrotation: A Rare Association

**DOI:** 10.1155/2012/354514

**Published:** 2012-08-08

**Authors:** Sonal Jain, Ragini Singh, Surendra Kumar Singh, Vikram Singh, Kumar Shantanu

**Affiliations:** ^1^Department of Radiodiagnosis, CSM Medical University (Erstwhile King George Medical University), Lucknow 226003, India; ^2^Department of Surgery, Fatima Hospital, Lucknow 226006, India; ^3^Department of Orthopedics, CSM Medical University (Erstwhile King George Medical University), Lucknow 226003, India

## Abstract

Abdominoscrotal hydrocele is an uncommon clinical entity and so is intestinal malrotation. We report a case of 15 year old boy who presented with lump in abdomen previously diagnosed as mesenteric cyst on ultrasound. A multislice CT scan and repeat ultrasound not only diagnosed the case as abdominoscrotal hydrocele but also detected intestinal malrotation with positive whirl sign. This is the first reported case of abdominoscrotal hydrocele with intestinal malrotation.

## 1. Background

Abdominoscrotal hydrocele (ASH) is an uncommon clinical entity, accounting for only 0.17% of all types of hydrocele [1]. ASH presents as a dumbbell-shaped giant hydrocele that occupies the scrotum and extends into the abdominal cavity through the inguinal ring.

The incidence of intestinal malrotation is 1 in 500 [2]. Malrotation is defined as any deviation from the normal 270° counterclockwise rotation of the bowel that occurs during embryogenesis. The resultant-shortened mesenteric pedicle predisposes to midgut volvulus, a clockwise rotation around the superior mesenteric artery axis that can lead to bowel ischemia.

This is the first reported case of ASH associated with intestinal malrotation.

## 2. Case Presentation

A 15-year-old boy came to the department of radiodiagnosis at our institute with the requisition for computed tomography (CT). The patient had history of lower abdominal swelling for the past one year, associated with a dull dragging pain. He had no history of fever or trauma. Ultrasound examination, performed elsewhere, showed a large cystic anechoic lesion in lower abdominal cavity suggesting mesenteric cyst.

On clinical examination, there was a mildly tender, soft lump in the right iliac fossa and hypogastric region measuring approximately 3 × 3 cm in size. There was no associated rise of local temperature.

**Figure 1:**
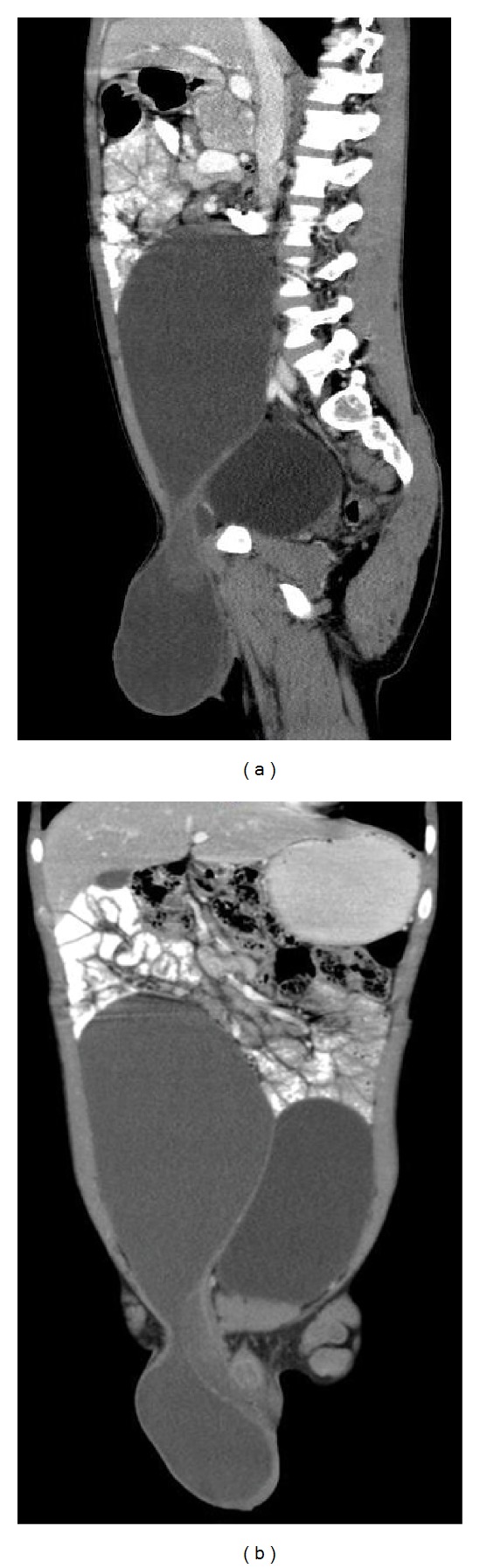
(a) and (b) Coronal and sagittal postcontrast CT images showing a large cystic lesion of thin fluid attenuation in right iliac fossa and lumbar region extending into right scrotal sac. Right testis is located in the right inguinal canal.

Multislice contrast-enhanced CT scan was done on Philips Brilliance TMCT scanner. A large cystic lesion of thin fluid attenuation was seen in right iliac fossa and lumbar region extending up to the umbilical region (Figures 1(a) and 1(b)). The lesion was extending into the right scrotal sac through the inguinal canal suggesting an abdominoscrotal hydrocele. Undescended right testis was seen, located within the inguinal canal in the region of superficial inguinal ring. A small hydrocele was also noted on the left side.

**Figure 2:**
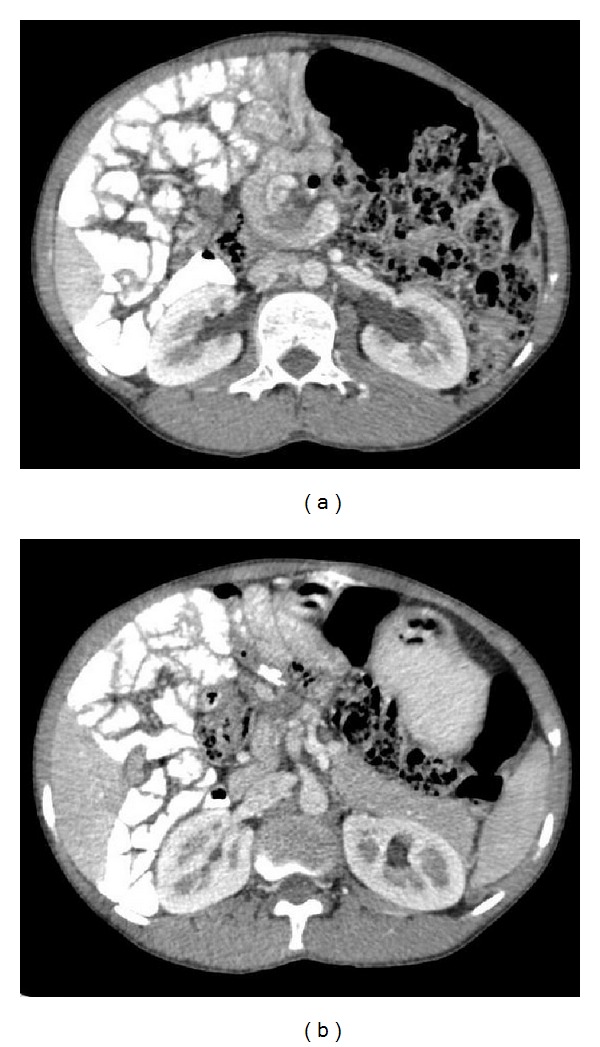
(a) Axial postcontrast CT image showing duodenum and SMV wrapping around axis of SMAin clockwise direction producing the characteristic whirl sign. Dilatation of SMV is also noted. Jejunal loops are seen of on right side of abdominal cavity. (b) Axial postcontrast CT image showing reversal of the normal anatomic relation of the SMAand SMV, that is, SMV is seen on the left side while SMA on the right side.

On further observation, duodenum and superior mesenteric vein (SMV) were seen wrapping around axis of superior mesenteric artery(SMA) in clockwise direction producing the characteristic whirl-like appearance (Figure 2(a)).

No obvious intestinal obstruction could be established. Furthermore, the jejunal loops were seen on right side of the abdominal cavity with reversal of the normal anatomic relation of the SMAand SMV that is; SMV was seen on left side while SMA on right side (Figure 2(b)).

**Figure 3:**
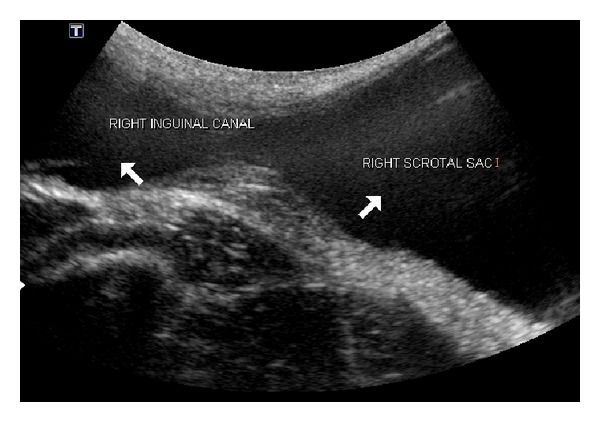
Ultrasound image at the level of right inguinal canal showing anechoic cystic lesion extending into scrotum and abdomen. Right testis is seen in the inguinal canal.

Ultrasound examination was repeated for the status of undescended testis. A large anechoic cystic lesion, measuring approximately 6 × 3 × 4 cm in size, in right iliac fossa extending inferiorly into right scrotum through the inguinal canal (Figure 3). Right testis was located within the inguinal canal. It was normal in size and echotexure with no evidence of neoplastic change.

On colour doppler examination, reversal of normal anatomic relation of SMV and SMA was seen (see Video 1 in Supplementary Materials available online at doi:10.1155/2012/354514). SMV and mesentery were seen twisting around SMA in clockwise direction producing the “Whirl pool” sign (see Video 2 in Supplementary Materials). Dilatation of SMV was also noted.

The patient was referred for surgery to the paediatric surgery department.

## 3. Discussion

Dupuytren first described ASH in 1834 as “hydrocele enbissac” (collections of fluid in the tunica vaginalis, which extends from the scrotum to the abdominal cavity) [3]. It is usually described in adult but in few reports it is also described in children. The etiology of development of ASH is controversial. The proposed pathogenesis of ASH is related to partial obliteration of the processus vaginalis, which serves as a one-way valve to “pump up” the scrotal portion of the hydrocele with intraperitoneal fluid during episodes of high intraabdominal pressure. At times, when the intrascrotal pressure exceeds the intraabdominal pressure, the proximal (intraabdominal) portion of the hydrocele expands, thus, the ASH becomes a dumbbell configuration with central constriction at the inguinal ring.

Ultrasound is usually adequate to confirm the diagnosis. Typically, ultrasound demonstrates encapsulated anechoic fluid collection extending from the abdomen to the scrotal cavity through an inguinal ring. However, if the relationship between the abdomen and the scrotal sac cannot be clearly delineated, then CT or magnetic resonance imaging (MRI) via the multiplanar approach would help to delineate the full extent of the ASH.

Malrotation of the intestines results when the intestinal rotation and fixation that occurs during pregnancy fails to occur. This normally happens in the 4thand 12thweeks of fetal life. In the 4thfetal week, the entire bowel is a straight tube with the superior mesenteric artery (SMA). During the course of pregnancy, the bowel rotates in place to the left of the SMA at the ligament of treitz.

Most people who are affected by malrotation show signs of the condition soon after birth; however, in a minority, malrotation is diagnosed long after infancy and is not manifested by the typical clinical sign of bilious vomiting. The presentation may be with atypical or chronic symptoms, such as failure to thrive or late onset of symptoms [4]. Patients with intestinal malrotation may sometimes be entirely asymptomatic.

Intestinal malrotation may lead to midgut volvulus, a potentially life-threatening condition.Most patients with small bowel volvulus can be identified on CT through detection of a whirl sign. Fisher [5] described the whirl sign as a CT finding of midgut volvulus corresponding to the whirlpool sign described on ultrasound. It occurs when afferent and efferent bowel loops rotate around a fixed point of obstruction, which results in tightly twisted mesentery along the axis of rotation. These twisted loops of bowel and branching mesenteric vessels create swirling strands of soft tissue attenuation within a background of mesenteric fat attenuation. The whirl sign is best appreciated when imaging is perpendicular to the axis of bowel rotation.

In our case, though a whirl sign was seen, we could not establish any obvious intestinal obstruction on CT. There are various studies made by Gollub et al. [6] showing that the whirl sign had a sensitivity of 64% and a PPV of 21% in the detection of volvulus.

In conclusion, ASH is a rare entity but its association with intestinal malrotation is even rarer. A combination of ultrasound and multislice CT helped us in diagnosing the case which was earlier misdiagnosed as mesenteric cyst.

## Supplementary Material

Video 1: Colour doppler examination shows reversal of the normal anatomic relation of the Superior mesentric artery (SMA) and vein (SMV). SMA is seen in blue color with pulsatile flow while SMV is seen in red colour.
Video 2: shows twisting of SMV around SMA in clockwise direction producing the “whirlpool sign”.
